# Molecular signatures of *Janthinobacterium lividum* from Trinidad support high potential for crude oil metabolism

**DOI:** 10.1186/s12866-021-02346-4

**Published:** 2021-10-20

**Authors:** Amanda C. Ramdass, Sephra N. Rampersad

**Affiliations:** Biochemistry Research Lab (Rm216), Department of Life Sciences, Faculty of Science and Technology, The University of the West Indies, St. Augustine, Trinidad and Tobago

**Keywords:** *Janthinobacterium lividum*, L-tryptophan oxidase, Lipase phylogeny, Violacein

## Abstract

**Background:**

*Janthinobacterium lividum* is considered to be a psychrotrophic bacterial species. For the first time in the literature, *J. lividum* strains were isolated from Trinidad presenting with atypical features - hydrocarbonoclastic and able to survive in a tropical environment.

**Methods:**

Identification of the Trinidad strains was carried out through 16S rRNA phylogenetic analysis. Gene-specific primers were designed to target the *VioA* which encodes violacein pigment and the *EstA*/*B* gene which encodes secreted extracellular lipase. Bioinformatics analyses were carried out on the nucleotide and amino acid sequences of *VioA* and *EstA*/*B* genes of the Trinidad *Janthinobacterium* strains to assess functionality and phylogenetic relatedness to other *Janthinobacterium* sequences specifically and more broadly, to other members of the *Oxalobacteraceae* family of betaproteobacteria.

**Results:**

16S rRNA confirmed the identity of the Trinidad strains as *J. lividum* and resolved three of the Trinidad strains at the intra-specific level. Typical motility patterns of this species were recorded. VioAp sequences were highly conserved, however, synonymous substitutions located outside of the critical sites for enzyme function were detected for the Trinidad strains. Comparisons with PDB 6g2p model from aa231 to aa406 further indicated no functional disruption of the *VioA* gene of the Trinidad strains. Phylogeny of the *VioA* protein sequences inferred placement of all *J. lividum* taxa into a highly supported species-specific clade (bs = 98%). EstA/Bp sequences were highly conserved, however, synonymous substitutions were detected that were unique to the Trinidad strains. Phylogenetic inference positioned the Trinidad consensus *VioA* and *EstA* protein sequences in a clearly distinct branch.

**Conclusions:**

The findings showed that the primary sequence of VioAp and EstA/Bp were unique to the Trinidad strains and these molecular signatures were reflected in phylogenetic inference. Our results supported chemotaxis, possible elective inactivation of *VioA* gene expression and secreted lipase activity as survival mechanisms of the Trinidad strains in petrogenic conditions.

**Supplementary Information:**

The online version contains supplementary material available at 10.1186/s12866-021-02346-4.

## Background

Diverse hydrocarbonoclastic bacteria, have demonstrated the capacity to degrade hydrocarbons [1, 2], and their biochemical and ecological features should be explored further [3, 4]. Some bacteria possess a contradictory response to these pollutants; they can mineralize chemical compounds for growth but, when aromatic compounds exceed toxicity thresholds, derangement of structural and functional properties of their membranes occurs followed by death [5]. In response to changing environments, motile bacteria can navigate through micro-environments to locate metabolizable compounds for growth and survival, and avoid sites of low nutrient availability, through complex metabolism-dependent and independent chemotaxis [6–12].

*Janthinobacterium lividum* belongs to the family *Oxalobacteraceae*. This family comprises 13 genera [13], whose membership consists of *J. lividum* [14], *J*. *svalbardensis* [15], and *J. agaricidamnosum* [16] as well as the recently reported *J*. *violaceinigrum* sp. nov., *J*. *aquaticum* sp. nov., and *J*. *rivuli* sp. nov. [17]. *Janthinobacterium* species are Gram-negative, motile, rod-shaped, strictly aerobic, chemoorganotrophic, and usually grow at a temperature optimum of 25-30 °C which is typical of mesophiles [13]; however, psychrophilic strains can grow at 4 °C [18]. Although *Janthinobacterium* strains have been isolated from soil [19–21], they are more commonly found in various aquatic habitats [18, 21–23], including the skin of amphibians [24, 25], and rainbow trout [26].

*J. lividum* colonies can be purple-violet in color as a result of production of the pigment, violacein [19, 27–29]. Biosynthesis of violacein, under favourable conditions, is enabled through activation of an operon, *VioABCDE,* whose expression is regulated by the type of carbon source [30]. The first committed step in violacein production is catalysed by the protein product of *VioA*, L-tryptophan oxidase [30–33]. Reports of non-pigmented and partly pigmented *C. violaceum*, and *J. lividum* strains also suggest that violacein production may not be a reliable taxonomic characteristic [34]. In general, pigments produced by bacteria are important for protection against other bacteria, ultraviolet radiation, oxidants, extreme temperature, and desiccation [35–38]. However, the specific role of violacein in *J. lividum* strains, and the reason for elective inactivation of the *Vio* operon in non-pigmented strains are unknown.

Among the microbial enzymes that can mineralize certain hydrocarbons (e.g. polycyclic aromatic hydrocarbons - PAHs), lipases are one of the largest groups of commercially important enzymes (ranked third after proteases and carbohydrates) due to their diversity and versatility [12, 39–44]. Microbes produce lipases (E.C.3.1.1.3 – triacylglycerol acyl hydrolases) that act at the interface generated by a hydrophobic lipid substrate in a hydrophilic aqueous medium. In the case of oil-contaminated environments, secreted lipases play a critical part in inducing lipolytic reactions of emulsified hydrocarbons at the lipid-water interface which assists in hydrocarbon uptake by adapted microbes [45–49].

In Trinidad, *J. lividum* has been isolated from several chronically polluted sites in the southern part of the island where petrogenic mud volcanoes, natural oil seeps and leaking above-ground oil conduit pipelines are common to the landscape [50]. Initial assessments pointed to identification of these Trinidad strains as *J. lividum* based on 16S rRNA sequence comparisons, specifically via the 16S rRNA Blast server. The Trinidad strains presented with atypical features not been previously cited in the literature i.e. their oil-contaminated natural habitat, low oxygen availability in such soils that must limit the survival of these strict aerobes, they were only isolated from crude oil-contaminated soil with temperatures at or above 45 °C, and were only recovered as the sole bacterial member of naturally-occurring consortia with different yeast species. But, in culture, these Trinidad strains are highly efficient at crude oil degradation [50]. There are no previous reports of *J. lividum* inhabiting oil-contaminated terrestrial environments or other literature that describe a high capability to metabolize crude oil. Additionally, the Trinidad strains did not produce violacein in culture, despite several attempts to induce biosynthesis based on the findings of Pantanella et al. [38]. All of these strain-specific features challenge some of the more common assumptions associated with characterising *J. lividum* species based on phenotype.

Based on the foregoing, we took a targeted gene approach to understanding how these Trinidad *J. lividum* strains survive. The phylogenetic relationships of these Trinidad strains with closely related Gram-negative members of the *Oxalobacteraceae* family were assessed based on 16S rRNA phylogeny. To understand the lack of pigmentation of the Trinidad strains, we designed species- and gene-specific primers that target the *VioA* gene, and conducted comparative analysis of the nucleotide (nt) and amino acid (aa) sequences of this gene with other *J. lividum* strains, and other violacein pigment-producing species. Further, to determine how survival is affected by the ability of the Trinidad strains to secrete lipase in the lipolytic catalysis of crude oil, we focused on the detection of the *EstA*/*B* gene in Trinidad *Janthinobacterium* isolates by designing species- and gene-specific primers, and analysed the nt and aa sequences of this gene compared with *EstA/B* of other strains and species. Motility of all strains from Trinidad was also assessed. The findings of this work provided the first molecular insight into atypical oil-degrading *J. lividum* strains from Trinidad.

## Results

### Motility of Trinidad *J. lividum* strains

A video demonstrating the motility of Trinidad *J. lividum* strains has been submitted to the journal.

### 16S rRNA analysis

**Fig. 1:**
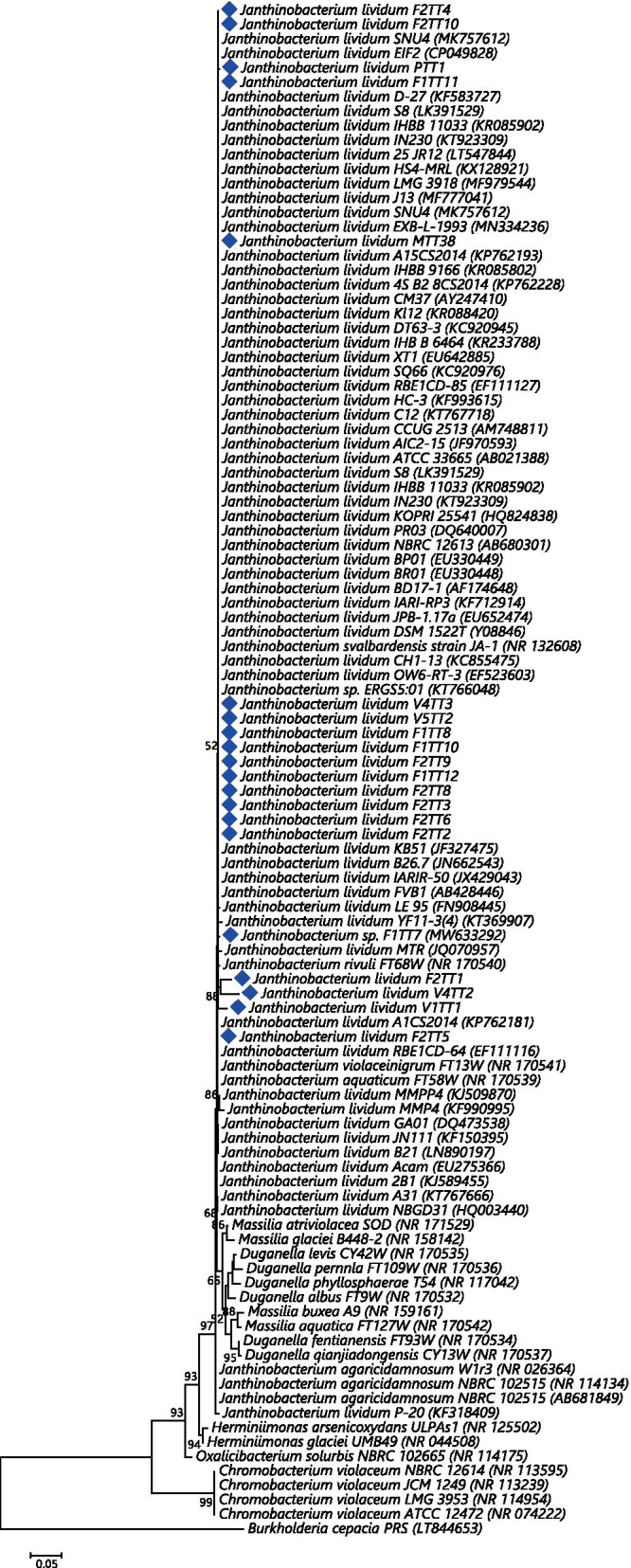
Molecular phylogenetic analysis by Maximum Likelihood method 16S rRNA. The evolutionary history was inferred by using the ML method based on the Jukes-Cantor model. The tree with the highest log likelihood (0.0000) is shown. The percentage of trees in which the associated taxa clustered together is shown next to the branches. Initial tree(s) for the heuristic search were obtained by applying the Neighbor-Joining method to a matrix of pairwise distances estimated using the Maximum Composite Likelihood (MCL) approach. A discrete Gamma distribution was used to model evolutionary rate differences among sites (6 categories (+G, parameter = 0.4823)). The tree is drawn to scale, with branch lengths measured in the number of substitutions per site. The analysis involved 106 nucleotide sequences. All positions containing gaps and missing data were eliminated. There were a total of 735 positions in the final dataset. Evolutionary analyses were conducted in MEGAX

The best substitution model (JC + G) was determined in MEGAX and the corresponding 50% bootstrap consensus Maximum Likelihood (ML) tree is presented (Fig. 1). The tree was rooted with *Burkholderia cepacia* strain PRS [34]. Trinidad strains were placed into the *J. lividum* clade. A sub-clade consisted of *Duganella* and *Massilini* members with moderate bootstrap support (bs = 65%). Some inter-specific sequence divergence was evident for members of the *Duganella* and *Massilini* clades. Distinct clades with high bootstrap support were obtained for *Oxalicibacterium*, *Chromobacterium*, and *Herminiimonas* species. High bootstrap support was also indicated for the *Chromobacterium* clade (bs = 99%). The *J. lividum* clade revealed polytomic branching for most taxa which was indicative of very low to no 16S rRNA sequence divergence for this species, and which confirmed the identity of the Trinidad isolates as *J. lividum* based on the assumption that sequences with 97% identity represent the same species [51]. 16S rRNA phylogenetic inference also revealed that placement of taxa was largely based on species, and there was no evidence of clustering according to geography or source of origin. 16S rRNA phylogeny was also able to resolve three (F2TT1, V4TT2 and V1TT1) out of the 20 Trinidad strains at the intra-specific level.

### *VioA* nucleotide and amino acid sequence analyses

**Fig. 2:**
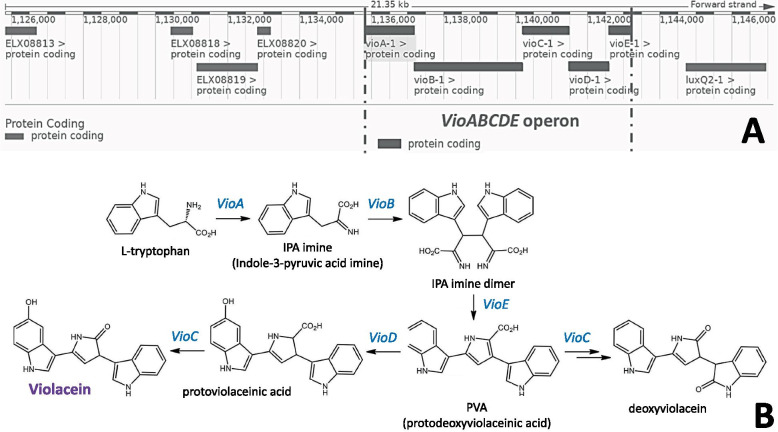
Violacein biosynthesis. **A** Biosynthetic gene cluster of *VioABCDE* operon of *Janthinobacterium lividum* GCA 900451145 (Gene ID: NCTC9796_03462) [56] that code for key enzymes in the five-step biosynthesis of violacein. **B** Biosynthetic pathway of violacein production. *VioA* encodes L-tryptophan oxidase which is the first enzyme required for IPA imine formation; this enzyme functions with an FAD co-factor. The *VioB* gene encodes an oxidase with a heme moiety, then utilizes IPA imine intermediate as a substrate to form the IPA imine dimer. The *VioE*-encoded enzyme in turn uses the dimer as a substrate, transforming it into intermediates for use by *VioD* and *VioC*-encoded enzymes. *VioD* and *VioC* encode FAD dependent-monooxygenases and both lead to the formation of violacein and deoxyviolacein

**Fig. 3:**
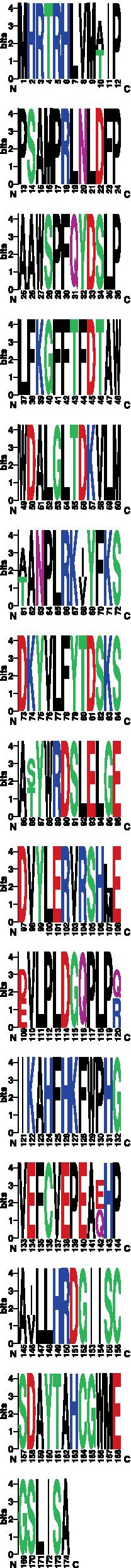
Weblogo illustration of aligned *VioA* amino acid sequences belonging to 40 different bacterial species including *VioA* consensus sequence of Trinidad strains

*VioA* must be activated to begin biosynthesis of violacein, and for this reason, the *VioA* gene of the *VioABCDE* operon (Fig. 2) was the focus of this study. Blastn and Blastp comparisons gave the highest probable identity of the nt and aa *VioA* sequences of the Trinidad strains as L-tryptophan oxidase which is an FAD-dependent amino-oxidoreductase. Sequence comparisons of the Trinidad strains as violacein-non-producers were carried out with a published violacein-producer (*J. lividum* strain JF266634 from Antarctica) [52, 53], and with a published *C. violaceum* strain ATCC 12472 [54, 55]. A high level of conservation was indicated by alignments of *VioA* aa sequences. The aligned *VioA* aa sequences are shown in Fig. 3.

**Fig. 4:**
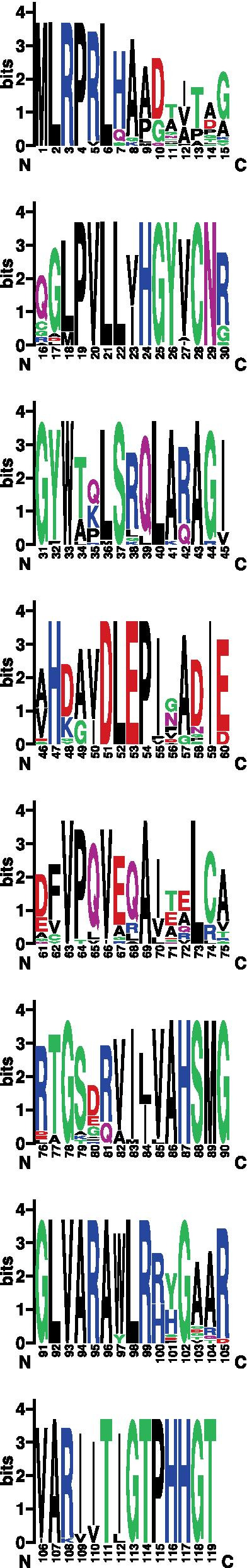
Weblogo illustration of aligned *VioA* amino acid consensus sequence of the Trinidad strains, non-violacein producer, with that of JF266634, violacein-producer

A comparison of the consensus aa sequence of the Trinidad strains (violacein non-producers) with violacein-producer *J. lividum* strain JF266634 revealed specific aa substitutions at positions outside of the FAD-binding site, outside of the L-tryptophan-binding site, and thus, outside the critical sites for enzyme function. The aa substitutions detected in the Trinidad consensus sequence were all predicted to be neutral according to analysis conducted on the PROVEAN (Protein Variation Effect Analyzer) server [57, 58], with a threshold predictor score > − 2.5. Further confirmatory analysis of these positions with mutagenesis studies of catalytic residues of *C. violaceum* strain ATCC 12472 revealed that all residues that are required for binding of the FAD cofactor and L-tryptophan substrate were absolutely conserved in the Trinidad strains. Hence, all critical sites were highly conserved. Two additional binding sites (located at aa16 and aa240) associated with Mg^2+^ metal binding as reported for *C. violaceum* strain ATCC 12472 in UniProtKB (Q9S3V1) [59], were also conserved for Trinidad strains, and for *J. lividum* JF266634. The aligned *VioA* aa consensus sequence of the Trinidad strains with that of JF266634 is shown in Fig. 4.

Examination of the PDB 6g2p model, which included 418 amino acid residues, revealed consensus of all binding sites in the sequences compared in this study. The Trinidad consensus sequence and JF266634 sequence aligned with the PDB model from aa231 to aa406. These analyses indicate no functional disruption of the *VioA* gene of the Trinidad strains.

### *VioA* protein tree

**Fig. 5:**
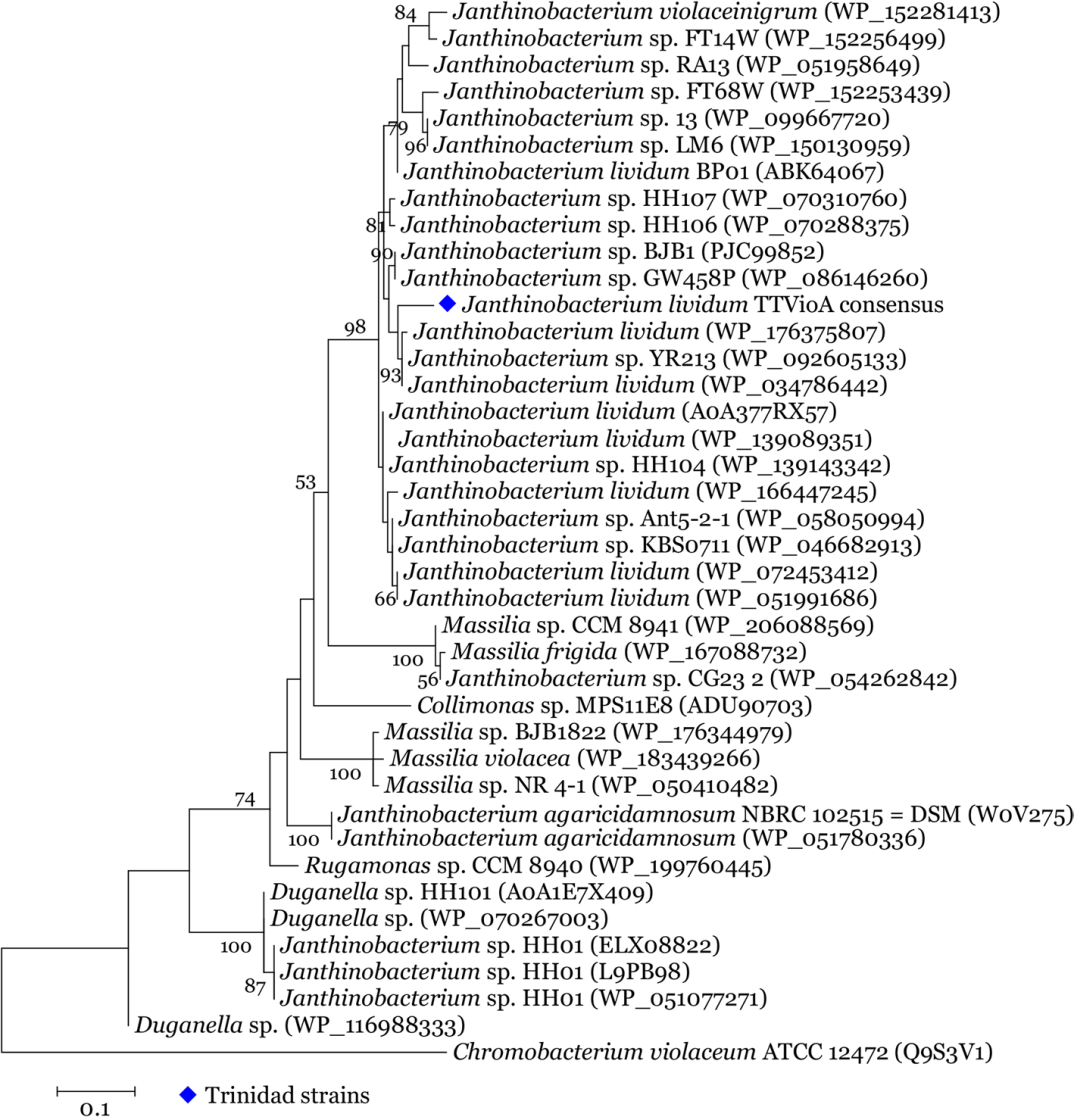
Molecular phylogenetic analysis by Maximum Likelihood method *VioA*. The evolutionary history was inferred by using the Maximum Likelihood method based on the JTT matrix-based model. The tree with the highest log likelihood (− 1780.4808) is shown. The percentage of trees in which the associated taxa clustered together is shown next to the branches. Initial tree(s) for the heuristic search were obtained by applying the Neighbor-Joining method to a matrix of pairwise distances estimated using a JTT model. A discrete Gamma distribution was used to model evolutionary rate differences among sites (6 categories (+G, parameter = 1.0012)). The tree is drawn to scale, with branch lengths measured in the number of substitutions per site. The analysis involved 40 amino acid sequences. All positions containing gaps and missing data were eliminated. There were a total of 161 positions in the final dataset. Evolutionary analyses were conducted in MEGAX

Deduced *VioA* protein sequences of all *J. lividum* taxa, including Trinidad consensus protein sequence, were placed into a highly supported clade (bs = 98%). Other highly supported clades were species-specific (Fig. 5).

### *EstA*/*B* (Alpha beta-hydrolase) nucleotide and amino acid sequence comparisons

The order of amino acids in the consensus protein sequence of the Trinidad strains formed structural domains indicative of hydrolytic enzymes with a characteristic alpha/beta fold. The ESTHER database (ESTerases and alpha/beta-Hydrolase Enzymes and Relatives) [60] also confirmed these features. The *EstA*/*B* protein sequences fold to produce “a catalytic triad”, which represent the highest conserved structural features of the fold. These enzymes have diverged from a common ancestor to preserve the arrangement of the catalytic residues, and not necessarily the binding site, which indicates substrate diversity for this enzyme class. Those aa sequences that lack one or all of the catalytic residues are, therefore, inactive. The aa sequence of the Trinidad strains belonged to the CATH Superfamily 3.40.50.1820 [56].

**Fig. 6:**
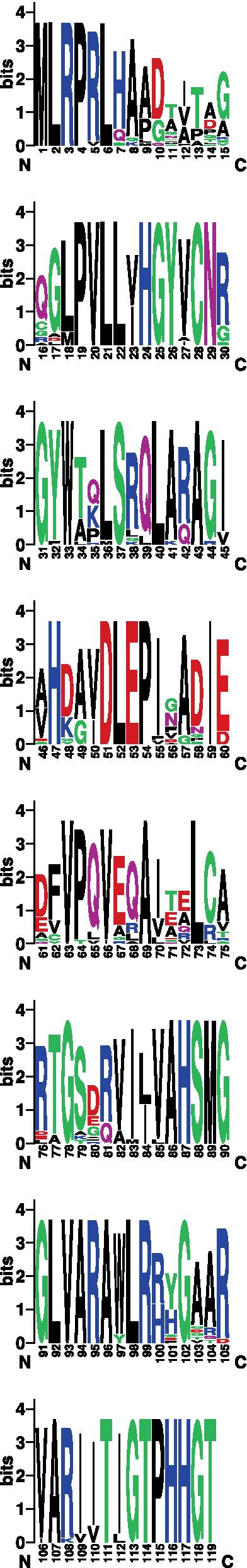
WebLogo 3 illustration of conserved and variable *EstA* amino acid positions in the aligned amino acid consensus sequences of the Trinidad strains and two other reference sequences

A comparison of 200 *EstA*/*B* aa sequences revealed high conservation, particularly one invariant block, “VAHSMGGANTL” located at aa74 to aa84, and which was present in all 200 reference sequences which included *Bacillus* species, and the consensus sequence of the Trinidad *J. lividum* strains. This block was also the longest length of conserved aa in the protein sequence. The conserved and variable aa positions in the consensus protein sequence of the Trinidad strains compared with two other reference sequences are illustrated in Fig. 6.

A search of UniProtKB confirmed the subcellular location of the consensus *EstA*/*B* aa sequence of the Trinidad strains as extracellular, and both the ESTHER and UniProtKB databases confirmed the presence of characteristic domain features of a signal peptide and transmembrane region which are both required for secretion of this enzyme to the extracellular environment.

PROVEAN indicated that the aa substitutions in the Trinidad *EstA*/*B* consensus sequence were predicted to be neutral based on a threshold variant score > − 2.5. Thus, the nt substitutions of the Trinidad strains were synonymous, and had no effect on the primary structure of the enzyme.

A comparison of the genomic region in Patric 3.6.9 (Pathosystems Resource Integration Center; https://www.patricbrc.org/) [61] indicated conserved genomic organization and synteny of this gene among six species that belonged to the *Janthinobacterium* genus. Regions that flanked this gene differed in sequence and gene product.

### *EstA* protein tree

**Fig. 7:**
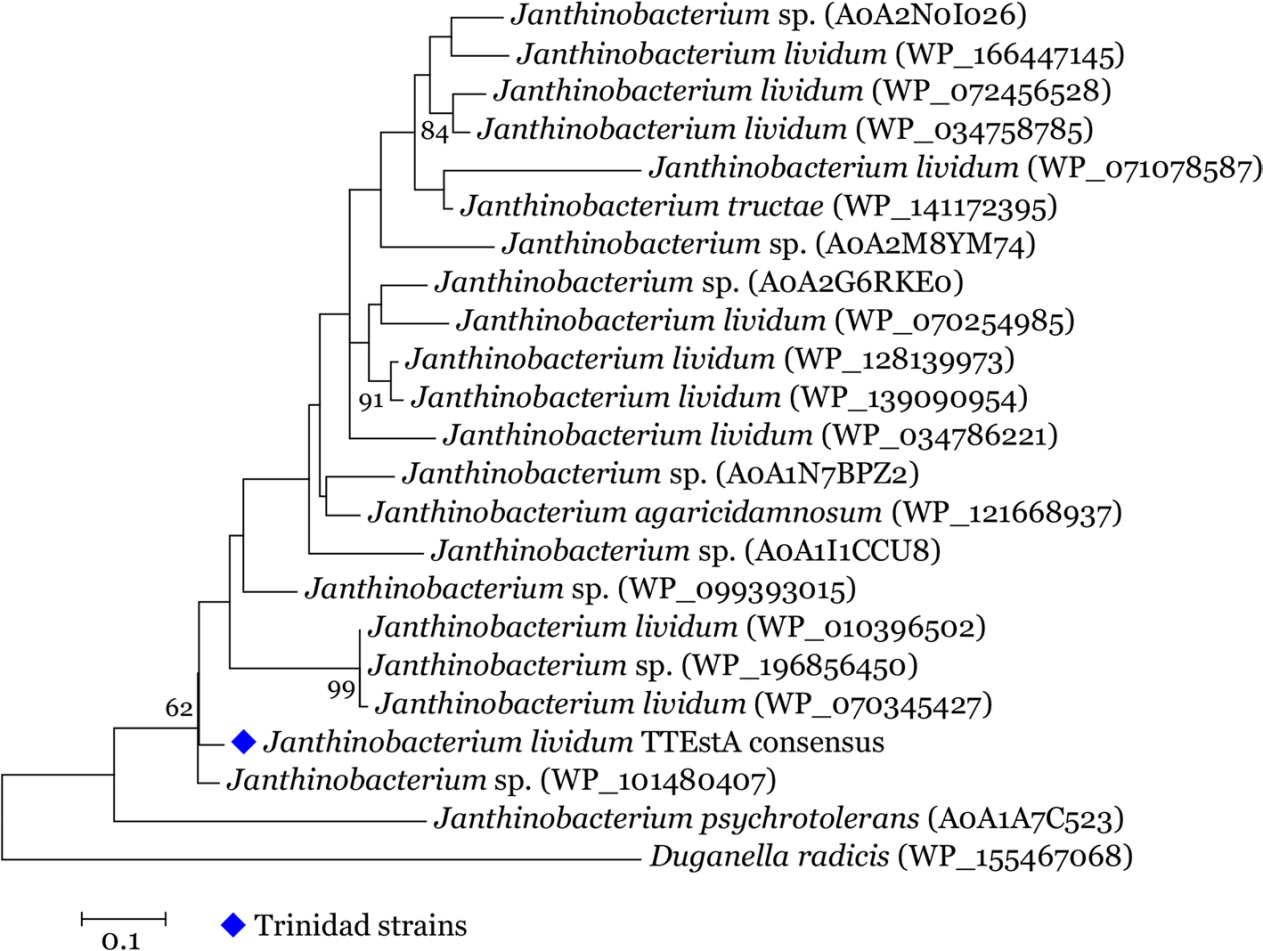
Molecular phylogenetic analysis by Maximum Likelihood method *EstA*. The evolutionary history was inferred by using the Maximum Likelihood method based on the JTT matrix-based model. The tree with the highest log likelihood (− 1566.2302) is shown. The percentage of trees in which the associated taxa clustered together is shown next to the branches. Initial tree(s) for the heuristic search were obtained by applying the Neighbor-Joining method to a matrix of pairwise distances estimated using a JTT model. A discrete Gamma distribution was used to model evolutionary rate differences among sites (6 categories (+G, parameter = 0.7842)). The tree is drawn to scale, with branch lengths measured in the number of substitutions per site. The analysis involved 23 amino acid sequences. All positions containing gaps and missing data were eliminated. There were a total of 116 positions in the final dataset. Evolutionary analyses were conducted in MEGAX

Phylogenetic inference based on deduced protein sequence of the *EstA* gene indicated aa variation for the majority of the taxa indicated by bifurcating branches, however, there was a single polytomic branch that consisted of three taxa which was highly supported (bs = 99%). The Trinidad consensus *EstA* protein sequence was positioned in a distinct branch with moderate boot strap support (62%) (Fig. 7).

## Discussion

16S rRNA phylogeny positioned the Trinidad *J. lividum* strains in a strongly-supported species-specific clade and sequence similarity among other *Janthinobacterium* species was above the recommended identity threshold. There was also polytomic branching among the *J. lividum* species indicative of low 16S rRNA sequence variation among of majority of those taxa belonging to this clade. The 16S rRNA gene sequence was, therefore, unable to resolve intraspecies variability. Similar findings were reported by [62–65]. Colony morphology or 16S rRNA gene sequence alone may not always accurately represent taxonomy of this species [66].

*J. lividum* is commonly isolated from aquatic and temperate environments [67] and as such, must be able to navigate and explore their habitats. Motility enables cells to sequester essential resources more efficiently and avoid low-nutrient areas in a highly competitive environment [68]. For the first time, *J. lividum* was isolated from terrestrial natural crude oil seeps and an asphaltic mud volcano in Trinidad and the strains were confirmed to be motile in this study. Motility in such terrestrial environments may be explained by the ability of this bacterial species to rely on gradient-guided swimming (chemotaxis) in a heterogeneous soil matrix [69]. Trinidad *J. lividum* strains inhabit non-water-saturated, petrogenic soil, and as such, have an adaptive need for energetically-expensive motility. Flagellar motility, including swarming [69], in unsaturated soils is restricted to saturation events, which are often short and sporadic e.g. rainfall and formation of rivulets that increase connectedness by creating micrometric aqueous films that may be thin and patchy. This would facilitate improved dispersal, and hence access to novel carbon sources but on a restricted scale. Motility can also lead to fitness gains conferred by fast surface colonization during transient wet periods which may, in part, explain the sustained presence of flagellated prokaryotes in partially saturated habitats such as soil surfaces [70].

Additionally, bacteria can disperse along fungal hyphae, which create “fungal highways” [71–78] through air spaces in unsaturated soil. Hydrophilic hyphae grow through air pockets in the soil and the liquid film present around hyphae allows bacteria to disperse over air-filled soil spaces [71, 75]. Bacterial motility via mycelium-associated dispersal helps to explain the persistence of flagellar motility in non-water-saturated environments [77], including oil-contaminated terrain [78].

The *VioA* gene of the *VioABCDE* operon was detected in non-violacein-producing Trinidad *J. lividum* strains. A non-violacein producing strain of *J. lividum* has also been reported by Kumar et al. [34], however, no *VioABCDE* operon was detected in whole genome sequence analysis. Friedrich et al. [79] described high nucleotide sequence conservation of the *VioABCDE* operon in a number of different *J. lividum* strains and reported conservation of this genomic block at the intraspecific level only. Our analyses indicated species-specific resolution with no evidence of functional disruption of the *VioA* gene product of the Trinidad strains. Similarly, Choi et al. [80] reported species-based placement of taxa for their *VioA* nucleotide sequence analysis. It is possible that the absence of violacein pigment in culture may be the result of operon inactivation due to missing induction signals [81]. Elective inactivation of this *VioA* gene is likely a consequence of co-metabolic synergy of Trinidad *J. lividum* strains with different yeast species for survival. Similar findings of multi-layered regulation of bacterial biosynthetic operons as an adapted regulatory response in relation to fluctuating, competitive and frequently stressful growth conditions were reported by Bervoets and Charlier [82].

The absence of violacein production in the Trinidad *J. lividum* strains can also be explained by the fact that they were only co-isolated with different species of yeast. Quorum sensing (QS) plays an important role in cross-kingdom signalling, and molecular dialogue in this specific type of microbial consortia [83–85]. The genes of the *VioABCDE* operon have all been identified as QS-dependent genes according to Haack et al. [23]. Based on the results of that study, the “janthinobacterial autoinducer (JAI-1)” affects the violacein biosynthesis in *J. lividum* strain HH01 [33] which in turn, affects cell-cell as well as intra- and inter-species communication. Absence of this JAI-1 signalling molecule reduces the level of inhibition of fungal growth. The Trinidad *J. lividum* strains existed in co-culture with different species of yeast in their natural habitat. Our findings suggest that it is worth investigating the genetic and biological basis of *J. lividum* strains that elect to repress expression of this operon as a co-metabolic mechanism to co-exist with different yeast species in oil-polluted sites.

Interaction networks among members of a specific microbial consortium are significant as it drives complex reactions that can lead to important behavioural shifts [86], which may explain the existence of non-violacein producers that possess the *VioABCDE* operon. Perhaps the Black Queen hypothesis [87] can be applied to explain (i) the need for motile Trinidad *Janthinobacterium* strains, and (ii) co-metabolic synergy with yeast which were always recovered as a co-culture in vitro, and which proved to be very difficult to separate into pure, single cultures. This relationship suggests that evolution drives microorganisms to lose essential functions when there is another co-existing species to perform them thereby generating a co-dependency for survival. Such species become “keystone species” where even though they are rare, they are extremely important to the ecosystem [87].

There is a paucity of research related to secretory lipase genes and lipase protein sequences of *J. lividum* strains. Kumar et al. [34] detected three copies of lipase-encoding genes in the genome sequence of a non-violacein-producing, cold- and high altitude-adapted *J. lividum* strain ERGS5:01 with demonstrated lipolytic activity in vitro. In this study, synonymous mutations were detected in the EstA/Bp sequence that were unique to the Trinidad strains. While protein function was not affected, these mutations may alter the structure or function of an mRNA where fitness becomes affected [88]. The findings of Agashe et al. [89] suggest that even single, but highly beneficial synonymous mutations in bacteria, can facilitate rapid evolution and adaptation to their environment. Lebeuf-Taylor et al. [90] reported that for *Pseudomonads*, synonymous mutations in a given gene can have highly variable fitness effects (deleterious and advantageous), and that these mutations may be more commonplace in nature than previously thought. This study identified synonymous mutations in the *VioA* and *EstA/B* genes of Trinidad *J. lividum* strains. Notwithstanding the specific mechanism(s) by which these synonymous mutations in the *VioA* and *EstA/B* genes of Trinidad *J. lividum* strains their fitness effects, how often and to what degree they contribute to adaptation, should be explored because *J. lividum* is of commercial interest [34].

*J. lividum* is a strict aerobe and yet, the Trinidad strains have the ability to subsist in hydrocarbon-polluted tropical soils that experience rapid redox fluctuations over short time frames, and which are prone to fluctuations in oxygen levels [91]. But, atypical behaviour by *J. lividum* strains appears to be related to ecological adaptation [23]. For strict aerobes such as *Janthinobacterium* in petrogenic environments, the use of oxygen is essential but often inadequate for aerobic degradation due to limited air permeability, and little to no oxygen in water-logged soil after heavy rainfall [91–98]. Metabolic chemistry fundamentally shifts where water meets sediment. Oxygen concentrations in such ecosystem can rapidly and totally be consumed as a result of a combination of high carbon availability, warm temperatures, abundant rainfall, and clay soils [95]. For bacteria inhabiting tropical soils, a considerable portion of the native community evolved physiological mechanisms to contend with these redox fluctuations [98]. Also, a possible defence for withstanding redox stress as a result of low oxygen, is the deployment of substrate storage mechanisms where bacteria store polyphosphates and volatile fatty acids for delayed use as energy sources similar to studies in aerobic-anaerobic activated sludge [95, 98]. Bacteria can shift their cellular metabolism to adjust to the rate and route of carbon source utilization, pathways of electron flow for oxidation-reduction balance, and their mechanisms of energy production through modulation of gene expression and protein activation in spite of anoxic conditions [91–98].

## Conclusions

Our findings showed that the primary sequence of VioAp and EstA/Bp were unique to the Trinidad strains and these molecular signatures were reflected in phylogenetic inference. Our results supported chemotaxis, possible elective inactivation of *VioA* gene expression and secreted lipase activity as survival mechanisms of the Trinidad strains. These complementary mechanisms are closely linked to co-metabolism with other microbes, and may occur as an elective expression of specific genes to accommodate mutualistic survival in polluted soil. Research that examines the bacterial quorum sensing gene cascade*, jqsA, qseC*, and *qseS* will improve our understanding of how the unique partnership of *J. lividum*-yeast consortium is co-ordinately regulated. Whole genome and transcriptome sequence analyses of the Trinidad strains would also identify other metabolic pathways that contribute to their survival in polluted environments which can be further explored for bioremediation of contaminated sites and potential discovery of enzymes and proteins of commercial value.

## Methods

### Materials

Crude oil was obtained from CARIRI - Caribbean Industrial (St. Augustine, Trinidad and Tobago). R2A media and nutrient agar were obtained from HiMedia Laboratories LLC (West Chester, PA, USA). Streptomycin, tetracycline, Tris HCl, EDTA, lysozyme, proteinase K, ethidium bromide, olive oil, and Rhodamine 6G solution were obtained from Sigma-Aldrich (St. Louis, MO, USA). Maxwell® 16 Cell DNA Purification kits used for DNA extraction, and GoTaq® Green Master Mix and Nuclease-Free water for PCR were obtained from Promega (Madison, WI, USA). Primers were from Integrated DNA Technologies (Coralville, IA, USA). The Thermal Cycler 2720 to perform PCR was from Thermo Scientific (USA), and the MiniBIS Pro System to view PCR products was a DNR Bio Imaging System (Neve Yamin, Israel).

### Isolation of culturable *Janthinobacterium* sp.

*Janthinobacterium* environmental strains, previously reported as efficient hydrocarbon degraders, were used for experimentation in this study. They were selected for sequencing and biochemical analysis due to their ability to grow on, and utilize crude oil as a unique carbon source. Recovered as a co-cultures with yeast, separation to obtain *Janthinobacterium* strains was carried out as previously described [50]. The bacterial strains were grown and maintained on R2A media.

### Motility

*Janthinobacterium* cultures in the log phase of growth were Gram stained and observed under a Brightfield microscope for shape, size and motility [99].

### DNA extraction, PCR, sequencing, and identification of microbes

Total genomic DNA (gDNA) from bacterial cultures was extracted. Bacterial isolates (pure isolates and those in co-culture) were grown on R2A supplemented with 50 mg/L each of streptomycin and tetracycline in the dark for 16 h or longer until growth was sufficient for extraction. Plates were flooded with 500-700 μL of TE buffer (10 mM Tris HCl, 1 mM EDTA, pH 8). The wash was collected and transferred to a 1.5 mL centrifuge tube and 100 μL of 50 mg/L each of lysozyme and proteinase K were added. The samples were incubated at 37 °C for 2 h in a water bath with occasional mixing by inversion. Immediately after incubation the entire sample content was transferred to Maxwell® 16 Cell DNA Purification kits, and gDNA was extracted according to the manufacturer’s protocol. DNA extracts were diluted 1:4, and this served as the working DNA concentration for Polymerase chain reaction (PCR) amplification. The 16S rRNA gene region (expected PCR product size ~ 1750 bp) was amplified by PCR with universal primer pair 8F [100] and 1492R [101]. PCR reaction conditions consisted of an initial denaturation of 5 min at 96 °C followed by 33 cycles of 30 s of denaturation at 95 °C, 30 s of annealing at 55 °C, 2 min of primer extension at 72 °C, followed by a final extension of 5 min at 72 °C.

The PCR mixture, 25 μL total volume, contained 12.5 μL of GoTaq® Green Master Mix, 0.5 μL (10 μM) of each primer, 6.5 μL of Nuclease-Free water, and 5 μL of DNA template. All PCR was performed on Thermal Cycler 2720. PCR products were visualized on a 1.5% agarose gel stained with ethidium bromide, and visualized under MiniBIS Pro System (data not shown). Where amplification failed, samples were processed once again from the first PCR. Samples producing amplicons were sent for purification and sequencing (MCLAB, San Francisco, CA, USA). Identification of sequences was performed using BLASTn (Basic Local Alignment Search Tool) [102] algorithm against the National Centre for Biotechnology Information (NCBI) GenBank database, specifically the NCBI 16S rDNA database.

### *VioA* and *EstA*/B gene detection

*VioA*-specific primers were designed to detect the presence of the *VioA* gene in the Trinidad *Janthinobacterium* sp. isolates. *VioA*-encoded amino oxidase catalyzes the first committed step in the biosynthesis of violacein and hence, was selected as a marker for the presence of the violacein operon and a functional *VioA* gene. Similarly, *EstA*/*B* α/β hydrolase enzyme-specific primers were designed to detect the presence of *EstA*/*B* gene encoding secreted extracellular lipase/esterase. The PrimerQuest**™** Tool of IDT DNA Technologies [103] was used to design both sets of primers (Table 1).

**Table 1:** Primer specifications designed to amplify the *VioA* and *EstA*/*B* genes of *Janthinobacterium* sp.

**Primer name**	**Primer orientation**	**5′ to 3′ Primer sequence**	**Start nucleotide position**	**Primer length**	**Tm**^**a**^	**% GC**	**PCR product/bp**
*VioA* gene-Janthino	Forward Primer	TCGAGTTCGTCAGCCATTAC	365	20	61.7	50	
	Reverse Primer	CTTCTTCTTCCGTCCGTTGA	1264	20	61.7	50	900
*EstA*/*B*-Janthino	Forward Primer	GTTGATGCTGCTGCAAGTG	24	19	61.9	52.6	
	Reverse Primer	TGTCGTGATGCGAATAGATCG	784	21	62.1	47.6	761

^a^*Tm* PCR annealing temperature

The PCR mixture, final volume 25 μL, for each reaction included 12.5 μL of GoTaq® Green Master Mix, 0.5 μL (10 μM) of forward and reverse primers, 6.5 μL of Nuclease-Free water, and 5 μL of DNA template. PCR reaction conditions included an initial denaturation at 94 °C for 5 min followed by 35 cycles of 94 °C for 1 min, 54 °C for 1 min, 72 °C for 1 min, ending with a final extension at 72 °C for 5 min. PCR reactions were carried out on Thermal Cycler 2720, and PCR products were examined on 1.5% agarose gels stained with ethidium bromide, and visualized under MiniBIS Pro System (data not shown). Samples producing amplicons were sent for purification and sequencing (MCLAB, San Francisco, CA, USA).

### *VioA* and *EstA*/*B* nucleotide and amino acid sequence analyses

The nt and aa sequences of *VioA* and *EstA*/*B* of the Trinidad *Janthinobacterium* strains were analysed to (i) confirm the identity of the gene sequences, and (ii) to assess the potential functionality of these genes and their associated gene products. Reference sequences were mined from GenBank [104] and UniProt [105], and an examination of gene regions of whole genomes in EnsemblGenomes [106] and EnsemblBacteria [107] were carried out. Information on violacein-producers and non-producers was recorded for each reference sequence. Translated gene products were predicted using the Expasy server [108]. aa sequence alignments were carried out in Clustal Omega [109]. A comparison of the aa substitutions of Trinidad strains with other strains was done using the PROVEAN server [57, 58], and protein secondary structures were compared on Phred software [110]. Weblogo 3 [111] was used to easily identify those specific aa positions for which there was high aa conservation and high aa variability. *VioA* gene features e.g. operon arrangement and domain synteny were examined in EnsemblBacteria [107]. Because of only minor differences in secondary structure prediction detected in Phred, PDB 3D model 6g2p (*C. violaceum* strain ATCC 12472) was used to assess the outcome of amino acid changes at critical sites in the protein model for L-tryptophan oxidase in terms of the FAD-cofactor binding sites, tryptophan substrate binding sites, and overall binding sites for this enzyme. This data was also compared to mutagenesis studies carried out for *C. violaceum* strain ATCC 12472 (UniProt Q953V1).

### Cluster analyses and phylogenetic inference

**Fig. 8:**
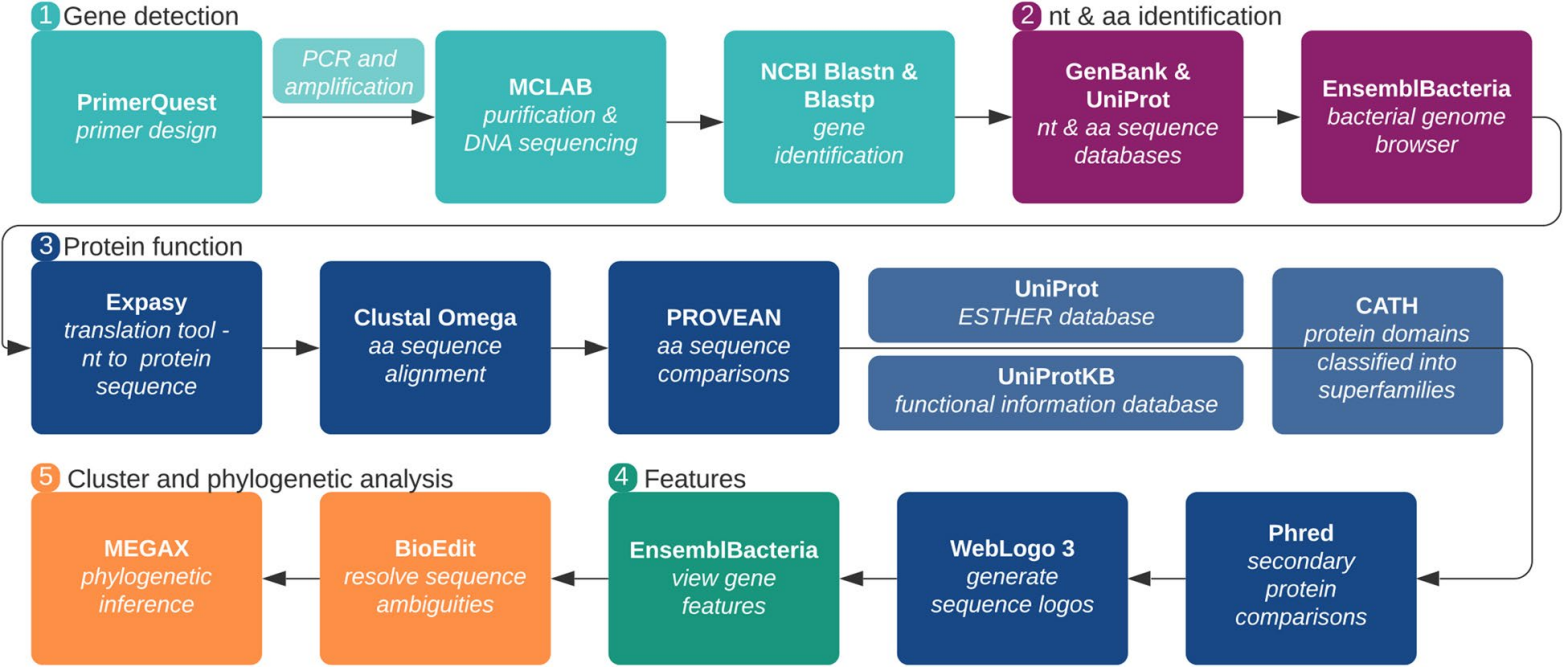
Bioinformatics workflow

16S rRNA, *VioA* and *EstA*/*B* aa sequences were analysed relative to sequences of other *Janthinobacterium* sequences specifically and more broadly, relative to other members of the *Oxalobacteraceae* family of betaproteobacteria. DNA sequences were manually edited using BioEdit software, version 7.1.9 [112] to resolve aa sequence ambiguities. Phylogenetic inference was carried out using the best fit model in MEGAX [113] using the ML algorithm with 1000 bootstrapped replications. The 50% consensus trees (bs > 50%) are presented. Reference sequence data are presented in Supplemental materials as Table S1 to Table S3. A summary of all bioinformatics analyses is presented in Fig. 8.

## Supplementary Information

**Additional file 1.**

**Additional file 2: Table S1.** List of reference sequences and GenBank accession numbers used in phylogenetic analyses of 16S rRNA gene region. **Table S2.** List of reference protein sequences and GenBank and UniProt accession numbers used in *VioA* protein tree. **Table S3.** List of reference protein sequences, GenBank and UniProt accession numbers used in construction of the *EstA*/*B* protein tree.

## Data Availability

All data generated or analysed during this study are included in this published article and its supplementary information files.
