# Supplementary material for: Molecular signatures of Janthinobacterium lividum from Trinidad support high potential for crude oil metabolism
Source: BMC Microbiol. 2021 Oct 20;21:287. doi: 10.1186/s12866-021-02346-4 (PMC8527658; doi:10.1186/s12866-021-02346-4)
Supplement: Supplementary file 2 — Additional file 2: Table S1. List of reference sequences and GenBank accession numbers used in phylogenetic analyses of 16S rRNA gene region. Table S2. List of reference protein sequences and GenBank and UniProt accession numbers used in VioA protein tree. Table S3. List of reference protein sequences, GenBank and UniProt accession numbers used in construction of the EstA/B protein tree. [file 12866_2021_2346_MOESM2_ESM.docx]

**Molecular Signatures of *Janthinobacterium lividum* from Trinidad Support High Potential for Crude Oil Metabolism**

**Supplemental Material**

**Supplemental Method**

**Induction of violacein production in culture**

This method is based on the report by Natalia et al. [77]. Once the identity of the bacterial strains was confirmed, culture conditions were varied in an attempt to induce violacein production by the Trinidad *Janthinobacterium* strains, as indicated by formation of purple-pigmented colonies. All attempts were unsuccessful, however, an outline of the methods used for induction is given below:

1. 1% to 10% glycerol added to medium which was Luria Broth (LB) broth and R2A semi-solid media
2. LB culture with agitation and without agitation at different temperatures 25°C, 37°C, 42°C
3. Antibiotic susceptibility disc assays involving the following antibiotics and their respective concentrations: aztreonam 30mcg/disc, imipenem-EDTA 10/750, kanamycin 30 mcg/disc, chloramphenicol 30 mcg/disc, ciprofloxacin 30 mcg/disc, trimethoprim 10 mcg/disc, ampicillin 10 mcg/disc, streptomycin 10 mcg/disc, erythromycin 15 mcg/disc and oxytetracycline 30 mcg/disc.

**Supplemental Tables**

Table S1. List of reference sequences and GenBank accession numbers used in phylogenetic analyses of 16S rRNA gene region.

| **Accession No.** | **Species** | **Strain** | **Origin** |
| --- | --- | --- | --- |
| MK757612 | *Janthinobacterium lividum* | SNU4 | South Korea |
| CP049828 | *Janthinobacterium lividum* | EIF2 | Germany |
| KF583727 | *Janthinobacterium lividum* | D-27 | China |
| LK391529 | *Janthinobacterium lividum* | isolate S8 | - |
| KR085902 | *Janthinobacterium lividum* | IHBB 11033 | India |
| KT923309 | *Janthinobacterium lividum* | IN230 | Austria |
| LT547844 | *Janthinobacterium lividum* | isolate 25_JR12 | - |
| KX128921 | *Janthinobacterium lividum* | HS4-MRL | Pakistan |
| MF979544 | *Janthinobacterium lividum* | LMG 3918 | Italy |
| MF777041 | *Janthinobacterium lividum* | J13 | - |
| MK757612 | *Janthinobacterium lividum* | SNU4 | South Korea |
| MN334236 | *Janthinobacterium lividum* | EXB-L-1993 | - |
| KP762193 | *Janthinobacterium lividum* | A15CS2014 | USA |
| KR085802 | *Janthinobacterium lividum* | IHBB 9166 | India |
| KP762228 | *Janthinobacterium lividum* | 4S_B2_8CS2014 | USA |
| AY247410 | *Janthinobacterium lividum* | CM37 | Germany |
| KR088420 | *Janthinobacterium lividum* | Kl12 | Germany |
| KC920945 | *Janthinobacterium lividum* | DT63-3 | China |
| KR233788 | *Janthinobacterium lividum* | IHB B 6464 | India |
| EU642885 | *Janthinobacterium lividum* | XT1 | China |
| KC920976 | *Janthinobacterium lividum* | SQ66 | China |
| EF111127 | *Janthinobacterium lividum* | RBE1CD-85 | Colombia |
| KF993615 | *Janthinobacterium lividum* | HC-3 | China |
| KT767718 | *Janthinobacterium lividum* | C12 | China |
| AM748811 | *Janthinobacterium lividum* | CCUG 2513 | - |
| JF970593 | *Janthinobacterium lividum* | AIC2-15 | Antarctica |
| AB021388 | *Janthinobacterium lividum* | ATCC 33665 | - |
| LK391529 | *Janthinobacterium lividum* | isolate S8 | - |
| KR085902 | *Janthinobacterium lividum* | IHBB 11033 | India |
| KT923309 | *Janthinobacterium lividum* | IN230 | Austria |
| HQ824838 | *Janthinobacterium lividum* | KOPRI 25541 | Austria |
| DQ640007 | *Janthinobacterium lividum* | PR03 | Austria |
| AB680301 | *Janthinobacterium lividum* | NBRC 12613 | - |
| EU330449 | *Janthinobacterium lividum* | BP01 | Alaska |
| EU330448 | *Janthinobacterium lividum* | BR01 | Alaska |
| AF174648 | *Janthinobacterium lividum* | BD17-1 | Korea |
| KF712914 | *Janthinobacterium lividum* | IARI-RP3 | India |
| EU652474 | *Janthinobacterium lividum* | JPB-1.17a | - |
| Y08846 | *Janthinobacterium lividum* | DSM 1522T | - |
| NR_132608 | *Janthinobacterium lividum* | JA-1 | Norway |
| KC855475 | *Janthinobacterium lividum* | CH1-13 | South Korea |
| EF523603 | *Janthinobacterium lividum* | OW6-RT-3 | Germany |
| KT766048 | *Janthinobacterium lividum* | ERGS5:01 | India |
| JF327475 | *Janthinobacterium lividum* | KB51 | Russia |
| JN662543 | *Janthinobacterium lividum* | B26.7 | Asia |
| JX429043 | *Janthinobacterium lividum* | IARI-R-50 | India |
| AB428446 | *Janthinobacterium lividum* | FVB1 | Japan |
| FN908445 | *Janthinobacterium lividum* | LE 95 | Spain |
| KT369907 | *Janthinobacterium lividum* | YF11-3(4) | China |
| MW633292 | *Janthinobacterium lividum* | F1TT7 | Trinidad and Tobago |
| JQ070957 | *Janthinobacterium lividum* | MTR | Chile |
| NR_170540 | *Janthinobacterium rivuli* | FT68W | China |
| KP762181 | *Janthinobacterium lividum* | A1CS2014 | USA |
| EF111116 | *Janthinobacterium lividum* | RBE1CD-64 | Colombia |
| NR_170541 | *Janthinobacterium violaceinigrum* | FT13W | China |
| NR_170539 | *Janthinobacterium aquaticum* | FT58W | China |
| KJ509870 | *Janthinobacterium lividum* | MMPP4 | India |
| KF990995 | *Janthinobacterium lividum* | MMP4 | India |
| DQ473538 | *Janthinobacterium lividum* | GA01 | - |
| KF150395 | *Janthinobacterium lividum* | JN111 | China |
| LN890197 | *Janthinobacterium lividum* | B21 | China |
| EU275366 | *Janthinobacterium lividum* | Acam | Peru |
| KJ589455 | *Janthinobacterium lividum* | 2B1 | China |
| KT767666 | *Janthinobacterium lividum* | A31 | China |
| HQ003440 | *Janthinobacterium lividum* | NBGD31 | India |
| NR_171529 | *Massilia atriviolacea* | SOD | China |
| NR_158142 | *Massilia glaciei* | B448-2 | China |
| NR_170535 | *Duganella levis* | CY42W | China |
| NR_170536 | *Duganella pernnla* | FT109W | China |
| NR_117042 | *Duganella phyllosphaerae* | T54 | - |
| NR_170532 | *Duganella albus* | FT9W | China |
| NR_159161 | *Massilia buxea* | A9 | China |
| NR_170542 | ‘*Massilia aquatica*’ Lu et al. 2020 | FT127W | China |
| NR_170534 | *Duganella fentianensis* | FT93W | China |
| NR_170537 | *Duganella qianjiadongensis* | CY13W | China |
| NR_026364 | *Janthinobacterium agaricidamnosum* | W1r3 | England |
| NR_114134 | *Janthinobacterium agaricidamnosum* | NBRC 102515 | - |
| AB681849 (outgroup) | *Janthinobacterium agaricidamnosum* | NBRC 102515 | - |
| KF318409 | *Janthinobacterium lividum* | P-20 | Kyrgyzstan |
| NR_125502 | *Herminiimonas arsenicoxydans* | ULPAs1 | - |
| NR_044508 | *Herminiimonas glaciei* | UMB49 | Greenland |
| NR_114175 | *Oxalicibacterium solurbis* | NBRC 102665 | - |
| NR_113595 | *Chromobacterium violaceum* | NBRC 12614 | - |
| NR_113239 | *Chromobacterium violaceum* | JCM 1249 | - |
| NR_114954 | *Chromobacterium violaceum* | LMG 3953 | unclear |
| NR_074222 | *Chromobacterium violaceum* | ATCC 12472 | Brazil |
| LT844653 (outgroup) | *Burkholderia cepacia* | PRS | Pakistan |
| **Trinidad strains** | |  |  |
|  | *Janthinobacterium lividum* | F2TT4 | Trinidad and Tobago |
|  | *Janthinobacterium lividum* | F2TT10 | Trinidad and Tobago |
|  | *Janthinobacterium lividum* | PTT1 | Trinidad and Tobago |
|  | *Janthinobacterium lividum* | F1TT11 | Trinidad and Tobago |
|  | *Janthinobacterium lividum* | MTT38 | Trinidad and Tobago |
|  | *Janthinobacterium lividum* | V4TT3 | Trinidad and Tobago |
|  | *Janthinobacterium lividum* | V5TT2 | Trinidad and Tobago |
|  | *Janthinobacterium lividum* | F1TT8 | Trinidad and Tobago |
|  | *Janthinobacterium lividum* | F1TT10 | Trinidad and Tobago |
|  | *Janthinobacterium lividum* | F2TT9 | Trinidad and Tobago |
|  | *Janthinobacterium lividum* | F1TT12 | Trinidad and Tobago |
|  | *Janthinobacterium lividum* | F2TT8 | Trinidad and Tobago |
|  | *Janthinobacterium lividum* | F2TT3 | Trinidad and Tobago |
|  | *Janthinobacterium lividum* | F2TT6 | Trinidad and Tobago |
|  | *Janthinobacterium lividum* | F2TT2 | Trinidad and Tobago |
|  | *Janthinobacterium lividum* | F2TT1 | Trinidad and Tobago |
|  | *Janthinobacterium lividum* | V4TT2 | Trinidad and Tobago |
|  | *Janthinobacterium lividum* | V1TT1 | Trinidad and Tobago |
|  | *Janthinobacterium lividum* | F2TT5 | Trinidad and Tobago |

Table S2. List of reference protein sequences and GenBank and UniProt accession numbers used in *VioA* protein tree.

| **Accession No.** | **Product** | **Source** |
| --- | --- | --- |
| WP_152281413 | FAD-dependent oxidoreductase | *Janthinobacterium violaceinigrum* |
| WP_152256499 | FAD-dependent oxidoreductase | *Janthinobacterium* sp. FT14W |
| WP_051958649 | FAD-dependent oxidoreductase | *Janthinobacterium* sp. RA13 |
| WP_152253439 | FAD-dependent oxidoreductase | *Janthinobacterium* sp. FT68W |
| WP_099667720 | FAD-dependent oxidoreductase | *Janthinobacterium* sp. 13 |
| WP_150130959 | FAD-dependent oxidoreductase | *Janthinobacterium* sp. LM6 |
| ABK64067 | putative tryptophan 2-monooxygenase VioA | *Janthinobacterium* lividum |
| WP_070310760 | FAD-dependent oxidoreductase | *Janthinobacterium* sp. HH107 |
| WP_070288375 | FAD-dependent oxidoreductase | *Janthinobacterium* sp. HH106 |
| PJC99852 | tryptophan oxidase | *Janthinobacterium* sp. BJB1 |
| WP_086146260 | FAD-dependent oxidoreductase | *Janthinobacterium* sp. GW458P |
| WP_176375807 | FAD-dependent oxidoreductase | *Janthinobacterium lividum* |
| WP_092605133 | FAD-dependent oxidoreductase | *Janthinobacterium* sp. YR213 |
| WP_034786442 | FAD-dependent oxidoreductase | *Janthinobacterium lividum* |
| A0A377RX57 | Probable L-tryptophan oxidase VioA | *Janthinobacterium lividum* |
| WP_139089351 | FAD-dependent oxidoreductase | *Janthinobacterium lividum* |
| WP_139143342 | FAD-dependent oxidoreductase | *Janthinobacterium* sp. HH104 |
| WP_166447245 | FAD-dependent oxidoreductase | *Janthinobacterium lividum* |
| WP_058050994 | FAD-dependent oxidoreductase | *Janthinobacterium* sp. Ant5-2-1 |
| WP_046682913 | FAD-dependent oxidoreductase | *Janthinobacterium* sp. KBS0711 |
| WP_072453412 | FAD-dependent oxidoreductase | *Janthinobacterium lividum* |
| WP_051991686 | FAD-dependent oxidoreductase | *Janthinobacterium lividum* |
| WP_206088569 | FAD-dependent oxidoreductase | *Massilia* sp. CCM 8941 |
| WP_167088732 | FAD-dependent oxidoreductase | *Massilia frigida* |
| WP_054262842 | FAD-dependent oxidoreductase | *Janthinobacterium* sp. CG23_2 |
| ADU90703 | putative tryptophan 2-monooxygenase | *Collimonas* sp. MPS11E8 |
| WP_176344979 | FAD-dependent oxidoreductase | *Massilia* sp. BJB1822 |
| WP_183439266 | FAD-dependent oxidoreductase | *Massilia violacea* |
| WP_050410482 | FAD-dependent oxidoreductase | *Massilia* sp. NR 4-1 |
| W0V275 | putative L-tryptophan oxidase VioA | *Janthinobacterium agaricidamnosum* NBRC 102515 = DSM 9628 |
| WP_051780336 | FAD-dependent oxidoreductase | *Janthinobacterium agaricidamnosum* |
| WP_199760445 | FAD-dependent oxidoreductase | *Rugamonas* sp. CCM 8940 |
| A0A1E7X409 | L-tryptophan oxidase VioA | *Duganella* sp. HH101 |
| WP_070267003 | FAD-dependent oxidoreductase | unclassified *Duganella* |
| ELX08822 | L-tryptophan oxidase VioA | *Janthinobacterium* sp. HH01 |
| L9PB98 | L-tryptophan oxidase VioA | *Janthinobacterium* sp. HH01 |
| WP_051077271 | FAD-dependent oxidoreductase | *Janthinobacterium* sp. HH01 |
| WP_116988333 | FAD-dependent oxidoreductase | unclassified *Duganella* |
| Q9S3V1 | Flavin-dependent L-tryptophan oxidase VioA | *Chromobacterium violaceum* ATCC 12472 |

Table S3. List of reference protein sequences, GenBank and UniProt accession numbers used in construction of the *EstA*/*B* protein tree.

| **Accession No.** | **Product** | **Source** |
| --- | --- | --- |
| A0A2N0I026 | triacylglycerol esterase/lipase *EstA* (alpha/beta hydrolase family) | *Janthinobacterium* sp. 64 |
| WP_166447145 | alpha/beta fold hydrolase | *Janthinobacterium lividum* |
| WP_072456528 | alpha/beta fold hydrolase | *Janthinobacterium lividum* |
| WP_034758785 | alpha/beta fold hydrolase | *Janthinobacterium lividum* |
| WP_071078587 | alpha/beta fold hydrolase | *Janthinobacterium lividum* |
| WP_141172395 | alpha/beta fold hydrolase | *Janthinobacterium tructae* |
| A0A2M8YM74 | triacylglycerol esterase/lipase *EstA* (alpha/beta hydrolase family) | *Janthinobacterium* sp. 67 |
| A0A2G6RKE0 | triacylglycerol esterase/lipase *EstA* (alpha/beta hydrolase family) | *Janthinobacterium* sp*.* 13 |
| WP_070254985 | alpha/beta fold hydrolase | *Janthinobacterium lividum* |
| WP_128139973 | alpha/beta fold hydrolase | *Janthinobacterium lividum* |
| WP_139090954 | alpha/beta fold hydrolase | *Janthinobacterium lividum* |
| WP_034786221 | alpha/beta fold hydrolase | *Janthinobacterium lividum* |
| A0A1N7BPZ2 | triacylglycerol esterase/lipase *EstA* (alpha/beta hydrolase family) | *Janthinobacterium* sp. TND4EL3 |
| WP_121668937 | alpha/beta fold hydrolase | *Janthinobacterium agaricidamnosum* |
| A0A1I1CCU8 | triacylglycerol esterase/lipase *EstA* (alpha/beta hydrolase family) | *Janthinobacterium* sp. 344 |
| WP_099393015 | alpha/beta fold hydrolase | *Janthinobacterium* sp. BJB446 |
| WP_010396502 | alpha/beta fold hydrolase | *Janthinobacterium lividum* |
| WP_196856450 | alpha/beta fold hydrolase | *Janthinobacterium* sp. CAN_S1 |
| WP_070345427 | alpha/beta fold hydrolase | *Janthinobacterium lividum* |
| A0A1A7C523 | triacylglycerol esterase/lipase *EstA* (alpha/beta hydrolase family) | *Janthinobacterium psychrotolerans* |
| WP_101480407 | alpha/beta fold hydrolase | unclassified *Janthinobacterium* |
| WP_155467068 | alpha/beta fold hydrolase | *Duganella radicis* |
